# Potential Gains in Life Expectancy Associated With Achieving Treatment Goals in US Adults With Type 2 Diabetes

**DOI:** 10.1001/jamanetworkopen.2022.7705

**Published:** 2022-04-18

**Authors:** Hamed Kianmehr, Ping Zhang, Jing Luo, Jingchuan Guo, Meda E. Pavkov, Kai McKeever Bullard, Edward W. Gregg, Naykky Singh Ospina, Vivian Fonseca, Lizheng Shi, Hui Shao

**Affiliations:** 1 Department of Pharmaceutical Outcomes and Policy, College of Pharmacy, University of Florida, Gainesville; 2 Division of Diabetes Translation, Centers for Disease Control and Prevention, Atlanta, Georgia; 3 Division of General Internal Medicine, University of Pittsburgh School of Medicine, Pittsburgh, Pennsylvania; 4 Center for Drug Evaluation and Safety (CoDES), University of Florida, Gainesville; 5 School of Public Health, Imperial College London, London, United Kingdom; 6 Division of Endocrinology, Diabetes, and Metabolism, University of Florida College of Medicine, Gainesville; 7 Department of Medicine and Pharmacology, School of Medicine, Tulane University, New Orleans, Louisiana; 8 Department of Health Policy and Management, School of Public Health and Tropical Medicine, Tulane University, New Orleans, Louisiana

## Abstract

**Importance:**

Improvements in control of factors associated with diabetes risk in the US have stalled and remain suboptimal. The benefit of continually improving goal achievement has not been evaluated to date.

**Objective:**

To quantify potential gains in life expectancy (LE) among people with type 2 diabetes (T2D) associated with lowering glycated hemoglobin (HbA_1c_), systolic blood pressure (SBP), low-density lipoprotein cholesterol (LDL-C), and body mass index (BMI) toward optimal levels.

**Design, Setting, and Participants:**

In this decision analytical model, the Building, Relating, Assessing, and Validating Outcomes (BRAVO) diabetes microsimulation model was calibrated to a nationally representative sample of adults with T2D from the National Health and Nutrition Examination Survey (2015-2016) using their linked short-term mortality data from the National Death Index. The model was then used to conduct the simulation experiment on the study population over a lifetime. Data were analyzed from January to October 2021.

**Exposure:**

The study population was grouped into quartiles on the basis of levels of HbA_1c_, SBP, LDL-C, and BMI. LE gains associated with achieving better control were estimated by moving people with T2D from the current quartile of each biomarker to the lower quartiles.

**Main Outcomes and Measures:**

Life expectancy.

**Results:**

Among 421 individuals, 194 (46%) were women, and the mean (SD) age was 65.6 (8.9) years. Compared with a BMI of 41.4 (mean of the fourth quartile), lower BMIs of 24.3 (first), 28.6 (second), and 33.0 (third) were associated with 3.9, 2.9, and 2.0 additional life-years, respectively, in people with T2D. Compared with an SBP of 160.4 mm Hg (fourth), lower SBP levels of 114.1 mm Hg (first), 128.2 mm Hg (second), and 139.1 mm Hg (third) were associated with 1.9, 1.5, and 1.1 years gained in LE in people with T2D, respectively. A lower LDL-C level of 59 mg/dL (first), 84.0 mg/dL (second), and 107.0 mg/dL (third) were associated with 0.9, 0.7, and 0.5 years gain in LE, compared with LDL-C of 146.2 mg/dL (fourth). Reducing HbA_1c _from 9.9% (fourth) to 7.7% (third) was associated with 3.4 years gain in LE. However, a further reduction to 6.8% (second) was associated with only a mean of 0.5 years gain in LE, and from 6.8% to 5.9% (first) was not associated with LE benefit. Overall, reducing HbA_1c_ from the fourth quartile to the first is associated with an LE gain of 3.8 years.

**Conclusions and Relevance:**

These findings can be used by clinicians to motivate patients in achieving the recommended treatment goals and to help prioritize interventions and programs to improve diabetes care in the US.

## Key Points

**Question:**

What potential gains in life expectancy (LE) are associated with lowering glycated hemoglobin (HbA_1c_), systolic blood pressure (SBP), low-density lipoprotein cholesterol (LDL-C), and body mass index (BMI) toward optimal levels in people with type 2 diabetes (T2D)?

**Findings:**

This decision analytical model using data from 421 adults with T2D showed that compared with individuals from the highest BMI, HbA_1c_, SBP, and LDL-C population quartile, those from the lowest BMI, HbA_1c_, SBP, and LDL-C population quartile had 3.9, 3.8, 1.9, and 0.9 years of additional LE, respectively.

**Meaning:**

These findings suggest that achieving recommended goals is likely to extend the LE of people with T2D.

## Teaser

This decision analytical model evaluates the gains in life expectancy associated with achieving treatment goals, including recommended body mass index,systolic blood pressure, and levels of glycated hemoglobin and low-density lipoprotein cholesterol, in US adults with type 2 diabetes.

## Introduction

People with type 2 diabetes (T2D) have increased risks of macrovascular and microvascular complications, which lead to an escalated risk of premature death.^1^ Compared with people without T2D at the age of 50, having T2D is associated with a life expectancy (LE) loss of 6 years.^2^ Better control of blood pressure, glucose and cholesterol levels, and body weight in people with T2D can potentially reduce the risk of diabetes-related complications and mortality, thus extending LE.^3,4,5^ Several studies have shown that higher body weight was associated with a substantial loss in LE.^6,7,8^ Other studies also found that lowering blood pressure among individuals with T2D could lead to a longer LE.^9,10,11^ The benefit from better management of glucose, blood pressure, cholesterol, and body weight is also associated with age and health conditions. A reduction of glycated hemoglobin (HbA_1c_; to convert to proportion of total hemoglobin, multiply by 0.01) from 8% to 6% was estimated to increase 1.2 life-years in women aged 55, but this benefit was much smaller in women in their 70s (0.8 life-years).^3^

Quantifying life-years gained from better diabetes care is imperative in clinical practice and designing public health interventions. Clinicians can use this information in the shared decision-making process with their patients, emphasizing the benefit of diabetes care in prolonging life expectancy. Policy makers can also use this information to prioritize and design public health interventions or programs. For the European population, LE associated with changes in glucose, blood pressure, cholesterol level, and body weight were estimated from the United Kingdom Prospective Diabetes Study (UKPDS)^3^ and the Framingham risk charts.^3,12,13^ Similar estimates are not available for the US population, a population distinct from Europe in terms of racial demographics, health care systems, and environment. Data have been lacking from long-term trials in the US to conduct the lifetime projection of individuals with T2D.

The completion of several long-term US-based trials (eg, the Action to Control Cardiovascular Risk in Diabetes [ACCORD] trial^14^) and recent innovation in the US-based diabetes simulation modeling provide essential data and methods needed for estimating the long-term benefit of improving diabetes care in the US. In this study, we used the Building, Relating, Assessing, and Validating Outcomes (BRAVO) diabetes model to quantify the potential gains in LE from achieving different levels of HbA_1c_, systolic blood pressure (SBP), low-density lipoprotein cholesterol (LDL-C), and body mass index (BMI; calculated as weight in kilograms divided by height in meters squared) in a representative population of US adults with T2D.

## Methods

This study was approved by the institutional review board at the University of Florida. Informed consent was waived because the data set is publicly available. The analysis was performed following the Consolidated Health Economic Evaluation Reporting Standards (CHEERS) reporting guideline. The overall goal of the study was to evaluate the association of modifiable biomarker control with LE in patients with T2D using a microsimulation experiment. To achieve this goal, we: (1) calibrated the validated BRAVO simulation model to a US nationally representative sample and (2) used the calibrated BRAVO model to evaluate LE in patients with T2D.

### Model Calibration

The BRAVO diabetes model is a discrete-time patient-level microsimulation model recently developed using data from the ACCORD trial.^14^ This model uses patients’ current risk profile to project long-term health outcomes, including diabetes-related complications, hypoglycemia, mortality, and the progression of modifiable biomarkers.^14,15^ Unlike most mainstream diabetes simulation models using the UKPDS risk equations, the BRAVO model uses novel risk equations developed from the ACCORD trial. The BRAVO diabetes model has been extensively validated and calibrated against international trials^15,16,17^ and used in several studies for program evaluation.^15,18,19^ Details of this model are provided in eFigure 2 in the Supplement.

Study participants enrolled in the ACCORD trial (2001-2009) were persons with T2D with elevated risks for cardiovascular complications. We performed model calibration to bridge the differences between ACCORD participants and the general T2D population in the US. To calibrate the BRAVO model for the general US population, we built a calibration sample from the 2009-2010 National Health and Nutrition Examination Survey (NHANES) and linked mortality records from the National Death Index.^20^ We then revalidated and recalibrated the BRAVO model using the NHANES calibration sample. We used relative bias (RB), defined as the ratio of the difference between projected and observed mortality to the observed mortality, to measure the projection accuracy of the model. Detailed methods are provided in eAppendix and eTable 1 in the Supplement.

### Study Population

We used the 2015 to 2016 NHANES survey cycle and corresponding survey weights to construct a US-representative study population of individuals with diabetes. The study included individuals aged 51 to 80 years with self-reported diagnosed diabetes by a clinician with data on demographic characteristics (age, race and ethnicity, duration of diagnosed diabetes, sex, education attainment, and current smoking status [yes or no]), and 4 measured biomarkers (HbA_1c_, SBP, LDL-C, and BMI). We excluded those with a self-reported history of cardiovascular disease as treatment goals and management strategies are likely to differ. Details regarding how information was collected in NHANES have been published previously.^21^

### Statistical Analysis

#### Gain in LE Associated With Biomarker Control at the Population Level

The BRAVO diabetes model uses the patients’ risk profile to populate the simulation of the diabetes progression and estimate the corresponding LE. As we focused on evaluating the potential life-year gain from multifaceted diabetes care in the US T2D population, we first grouped all persons with T2D into quartiles based on the distributions of the 4 biomarkers examined in our study. We then estimated the LE for patients from each quartile and the LE if patients from each quartile improved 1 of their biomarker levels to the next lower quartile while keeping other biomarkers constant. This allowed us to estimate the potential gains in LE associated with improvement of each biomarker from fourth to third, second, and first quartile, respectively.

#### Gain in LE Associated With Improved Biomarkers at the Individual Level

We further examined how an improvement in each biomarker is associated with LE at the individual level by age, sex, and levels of biomarkers. We grouped our simulation sample into 6 subgroups based on age (51-60 years, 61-70 years, and 71-80 years) and sex (male or female). Multiple alternative scenarios with different levels of each biomarker were simulated for each age-sex subgroup. Three BMI levels were tested: 25, 30, and 35. HbA_1c_was tested in three levels: 7%, 8%, and 10%. Four SBP levels (120 mm Hg, 140 mm Hg, 160 mm Hg, and 180 mm Hg) and 3 LDL-C levels (70 mg/dL, 100 mg/dL, and 130 mg/dL; to convert to millimoles per liter, multiply by 0.0259) were also examined according to the relevance of these risk levels in clinical practice.^3,22,23^ A heatmap was created to summarize the results from these simulation scenarios.

Although biomarker progressions after treatment can vary substantially across subgroups and be associated with patient characteristics (eg, treatment adherence, age) and external exposures (eg, access to care, environment), we assumed that once the goal is set, patients will adjust their treatment and put forth constant effort for achieving the goal. The improvement in biomarkers, both at the population and individual level, was assumed to sustain over time in our simulation. Thus, our estimation was the “ceiling effect” of goal achievement, which measured the benefit of LE extension when the biomarkers were kept under the goal throughout the lifetime window. SAS statistical software version 9.4 (SAS Institute) was used to perform the statistical analysis. Data were analyzed from January to October 2021.

## Results

We identified 421 individuals with T2D from the National Health and Nutrition Examination Survey (2015-2016). The mean (SD) age of the study sample was 65.6 (8.9) years, and 46% (194) were women. Detailed demographics and risk profiles of the target population are provided in eTable 3 in the Supplement. We grouped HbA_1c_ into quartiles (<6.4%, 6.4%-7.2%, 7.3%-8.2%, and >8.2%) with a mean of 5.9%, 6.8%, 7.7%, and 9.9% for each corresponding quartile. We grouped SBP into quartiles (<122 mm Hg, 122-132 mm Hg, 133-144 mm Hg, >144 mm Hg) with a mean of 114.1 mm Hg, 128.2 mm Hg, 139.1 mm Hg, and 160.4 mm Hg for each quartile respectively. We grouped LDL-C into quartiles (<73 mg/dL, 73-96 mg/dL, 97-122 mg/dL, >122 mg/dL) with a mean of 59 mg/dL, 84 mg/dL, 107 mg/dL, and 146.2 mg/dL for each quartile. BMI quartiles (<27, 27-31, 32-36, >36) had means of 24.3, 28.6, 33.0, and 41.4, respectively.

**Figure 1.**
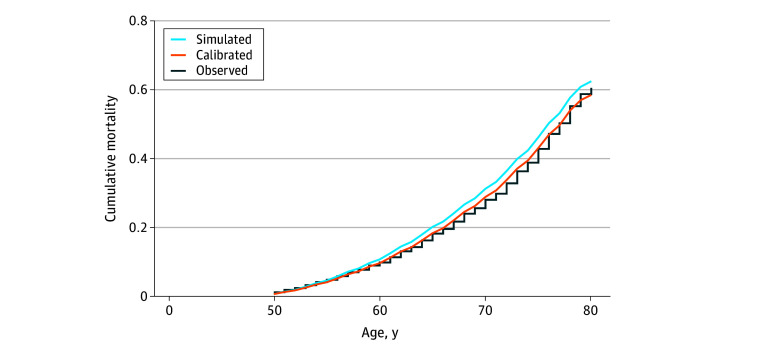
Cumulative Mortality Over 30 Years in Individuals With Type 2 Diabetes at Age 51 to 55 Years

Figure 1 presents the cumulative mortality over time for individuals aged 51 to 55 years. The solid line denotes the Kaplan-Meier curve for the observed cumulative mortality, serving as the benchmark for the calibration. This curve is created based on the observed annual mortality rate at each age year and no attrition during the follow-up. Details for this approach can be found in the eAppendix in the Supplement. The dashed line shows the projected cumulative mortality generated from the uncalibrated BRAVO model. The dotted line denotes the cumulative mortality projected using the calibrated BRAVO model. The BRAVO diabetes model produced projections with a mean 5-year RB of 13.3% over the 6 age groups before the calibration. The mean RB was reduced to 2.9% as a result of the calibration. Detailed measurements for model performance and interpretation were provided in eTable 2 in the Supplement.

**Figure 2.**
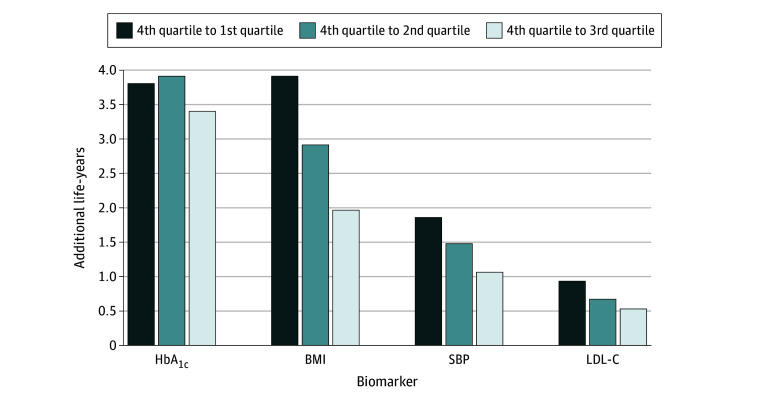
Gains in Life-Years Associated With Different Levels of Biomarkers in Individuals With Type 2 Diabetes The mean values of biomarkers for the first, second, third, and fourth quartile were as follows: glycated hemoglobin (HbA_1c_), 5.9%, 6.8%, and 7.7% vs 9.9% (to convert to proportion of total hemoglobin, multiply by 0.01); systolic blood pressure (SBP), 114.1 mm Hg, 128.1 mm Hg, and 139.1 mm Hg vs 160.4 mm Hg; low-density lipoprotein cholesterol (LDL-C), 58.9 mg/dL, 84.0 mg/dL, and 107.0 mg/dL vs 146 mg/dL (to convert to mmol/L, multiply by 0.0259), and body mass index (BMI, calculated as weight in kilograms divided by height in meters squared), 24.3, 28.6, and 33.0 vs 41.4.

Figure 2 presents gains in LE associated with improving biomarkers in people with T2D at a population level. For glucose control, reducing HbA_1c_ from 9.9% (mean of fourth quartile) to 7.7% (mean of third quartile) was associated with a mean of 3.4 years gain in LE. However, a further reduction from 7.7% to 6.8% (mean of second quartile) was associated with only a mean of 0.5 years gain in LE, and from 6.8% to 5.9% (mean of first quartile) resulted in no benefit in prolonging LE (0.1 years shorter in LE). Overall, reducing HbA_1c_ from Q4 to Q1 is associated with an LE gain of 3.8 years. Compared with individuals with a BMI level of 41.4 (mean of fourth quartile), a lower BMI level of 24.3 (mean of first quartile), 28.6 (mean of second quartile), and 33.0 (mean of 3rd quartile), were associated with a mean of 3.9, 2.9, and 2.0 additional life-years, respectively. Compared with individuals with an SBP level of 160.4 mm Hg (mean of fourth quartile), having a lower SBP level of 114.1 mm Hg (mean of first quartile), 128.2 mm Hg (mean of second quartile), and 139.1 mm Hg (mean of third quartile) were associated with 1.9, 1.5, and 1.1 years gain in LE, respectively. A lower LDL-C level of 59 mg/dL (mean of first quartile), 84.0 mg/dL (mean of second quartile), and 107.0 mg/dL (mean of third quartile) was associated with a mean of 0.9, 0.7, and 0.5. additional life-years, compared with individuals with LDL-C of 146.2 mg/dL (mean of fourth quartile), respectively. Because of the public health relevance, we also examined the benefit of smoking cessation, which ranged from 0.7 years for women aged 50 to 60 years to 1.1 years for men aged 70 to 80 years (eFigure 1 in the Supplement). LEs associated with different levels of biomarkers were presented more granularly in eFigure 3 in the Supplement. Risk reductions in diabetes-related complications associated with goal achievements were provided in eTable 4 in the Supplement.

**Figure 3.**
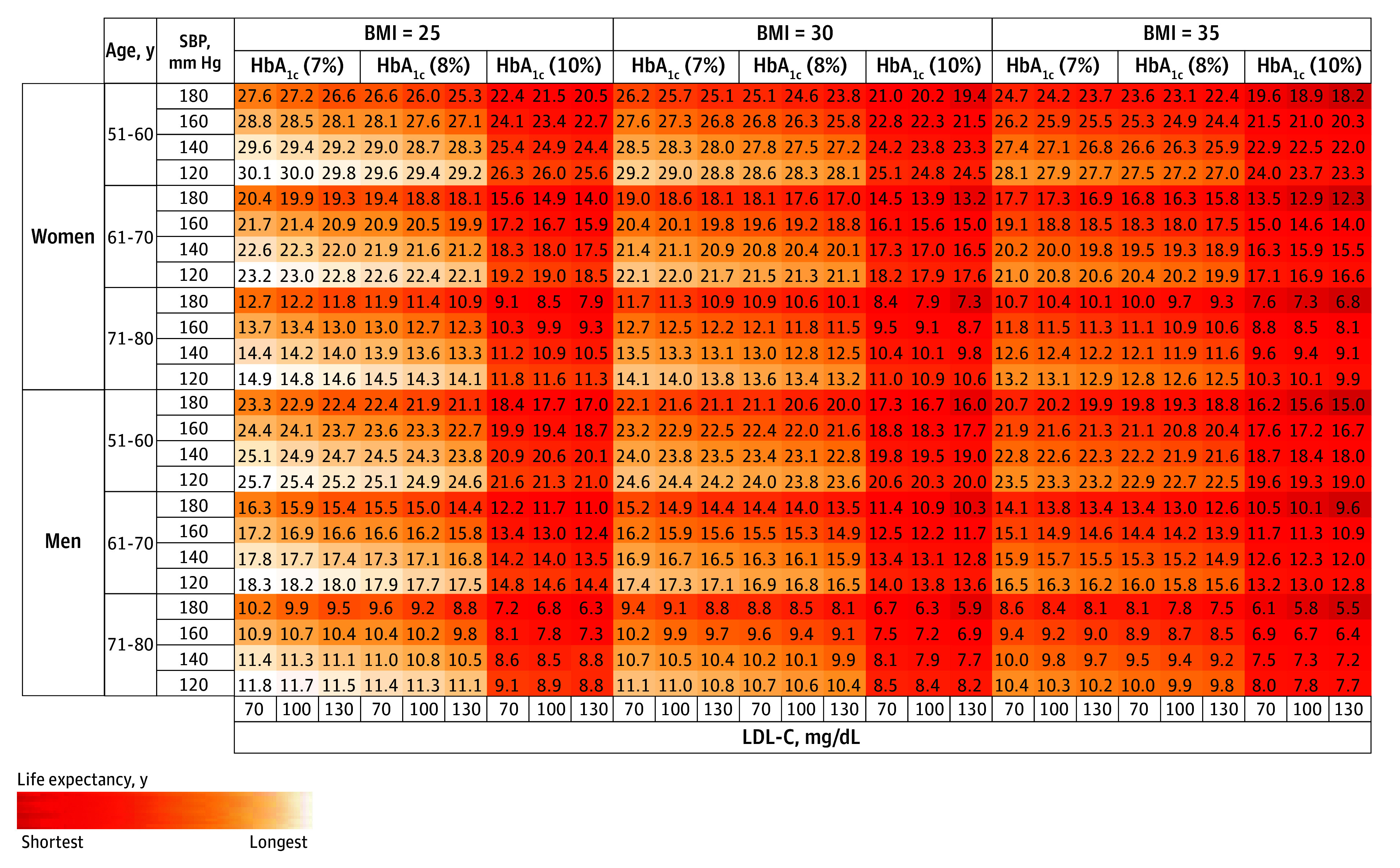
The Estimated Remaining Life-Years in Men and Women With Type 2 Diabetes and Without Cardiovascular Diseases The life expectancies are color-coded for each age-sex group separately. BMI indicates body mass index (calculated as weight in kilograms divided by height in meters squared); HbA_1c_, glycated hemoglobin (to convert to proportion of total hemoglobin, multiply by 0.01); LDL-C, low-density lipoprotein cholesterol (to convert to mmol/L, multiply by 0.0259); SBP, systolic blood pressure.

Figure 3 presents a heat map illustrating the LEs of individuals with T2D by different ages, sex, and biomarker levels. Red denotes high mortality risk (low LE), and white denotes low mortality risk (high LE). Estimated LEs in the lowest (lower left) and highest (upper right) risk groups ranged from 30.1 to 18.2 years in women aged 50 to 60 years, 23.2 to 12.3 years in women aged 60 to 70 years, and 14.9 to 6.8 years in women aged 70 to 80 years. Among men with T2D, LEs ranged from 15.0 to 25.7 years in those aged 50 to 60 years, 9.6 to 18.3 years in those aged 60 to 70 years, and 5.5 to 11.8 years in those aged 70 to 80 years. For example, the LE heatmap presented in Figure 3 suggests that a woman aged 50 to 60 years old with BMI 30, SBP 160 mm Hg, and HbA_1c_ 10% can expect to live an additional 3.0 years by reducing her SBP to 120 mm Hg, and can gain 1.2 years through reducing BMI to 25. For a male patient aged 50 to 60 years with BMI 35, SBP 160 mm Hg, HbA_1c_ 8%, and LDL-C 130 mg/dL, reducing BMI from 35 to 30 was associated with an additional 1.4 years of LE. However, for a male patient aged 70 to 80 with the same levels of biomarkers, reducing BMI to 30 kg/m^2^ was only associated with an additional 0.6 years of LE.

## Discussion

This study quantified the potential gains in LE associated with different levels of biomarkers in patients with diabetes. Differences in HbA_1c_ and BMI were found to have the strongest association with LE gain from a population perspective. At the individual level, we observed a large variation in the benefits associated with better diabetes care, associated with patients’ individual characteristics. The benefit of biomarker control was most pronounced in younger adults, and diminished as people aged. Better control of biomarkers can potentially increase the LE by 3 years in an average person with T2D in the US. For individuals with very high levels of HbA_1c_, SBP, LDL-C, and BMI, controlling biomarkers can potentially increase LE by more than 10 years.

LE gained through lower BMI was the largest among the 4 modifiable biomarkers we examined. For people with T2D and a high BMI (the fourth quartile, BMI >36), reducing their BMI to below 25 (the first quartile) was associated with an estimated LE gain of 3.9 years. This benefit, however, might not be easily achieved in clinical practice because it requires a substantial reduction in BMI. Our estimation can be referred to as a “ceiling effect,” which measures the potential LE gains under an optimal scenario. In clinical practice, patients can achieve halfway and gain a proportion of the estimated LE benefit, which is still estimated to be substantial. Thus, body weight reduction among persons with diabetes and obesity continues to be a clinical and public health priority.

Lifestyle interventions and medical nutrition therapies^24,25^ are effective in reducing body weight. In the Look AHEAD study,^26^ the intensive lifestyle intervention reduced body weight in people with T2D and overweight or obesity by 8.2% in the first year. When administered in a population with diabetes duration less than 3 years, intensive lifestyle intervention resulted in a 12% body weight reduction.^27^ Although the Look AHEAD study did not find a significant reduction in mortality associated with intensive weight control, we believe this was mainly attributable to the fact that Look AHEAD participants were relatively younger and had a shorter duration of diabetes and lower cardiovascular risks than the general population, in which a significant difference requires a much larger sample size and longer follow-up time to detect. Our estimated gain in LE was mainly associated with potential reductions in cardiovascular diseases associated with weight loss. According to data from ACCORD, a lower BMI level was associated with lower risks of congestive heart failure, angina, and revascularization, which in turn was associated with a lower risk of mortality. Bariatric surgery can lead to more than 25% body weight reduction.^28^ It has been reported that after bariatric surgery, a substantial proportion of patients have diabetes remission and stop using glucose-lowering medication.^28^ On the basis of our estimates, a 12% weight loss from lifestyle intervention was associated with an LE gain of approximately 1.5 years, and a 25% weight loss from bariatric surgery 3.0 years. This, however, assumes that weight loss can be maintained throughout the lifetime, which has been challenging for both lifestyle intervention and bariatric surgery.^29,30,31^ Maintaining weight loss is especially challenging for those receiving pharmacological therapy, as several commonly used glucose-lowering drugs, such as thiazolidinediones and insulin, are associated with weight gain.^32^ Newer treatments such as glucagon-like peptide 1 receptor agonists and sodium-glucose cotransporter-2 (SGLT2) inhibitors, however, have been shown to reduce body weight. Such features might be especially valuable for patients with BMI at the fourth quartile who can benefit most substantially from weight loss.

The potential gain of LE associated with HbA_1c_ control diminished as HbA_1c_ approached normoglycemia. Reducing HbA_1c_ from 9.9% (the fourth quartile) to 7.7% (the third quartile) was associated with a substantial increase in LE (ie, a mean of 3.4 years). However, not much additional LE accumulated from further reducing the HbA_1c_. This finding was consistent with the ACCORD trial, in which the intensive glycemic group (HbA_1c_ target <6.0%) had escalated mortality rates compared with the control group (HbA_1c_ target 7%-8%). A similar pattern was also observed from a previous study^33^ which found that the benefit was reduced when HbA_1c_ was decreased from 7.0% to normoglycemic level. However, the BRAVO diabetes model used ACCORD trial data to formulate its calculation algorithm, so it is not unexpected that these estimations agree with ACCORD findings. Some have suggested that the escalated mortality rate observed in the ACCORD treatment group was not attributable to the low HbA_1c_ target, but rather to how the low level of HbA_1c_ was achieved.^34^ Since only a small proportion of ACCORD participants used glucagon-like peptide 1 receptor agonists, and none used SGLT2 inhibitors to achieve the intensive glycemic goal, whether achieving intensive goals through these newer drugs can produce different results from the ACCORD trial is of great interest to the diabetes community. However, as we await such evidence, our study highlights the importance of controlling HbA_1c_ levels between 7.0% and 8.0%.

Lowering SBP from the fourth to first quartile was associated with a smaller change in LE compared with lowering BMI. However, this does not imply that SBP control is less important than BMI reduction. Our population-level estimates presented in Figure 2 are not designed for clinicians to prioritize 1 treatment over the other because treatment outcomes varied substantially based on patients’ individual characteristics. For example, the LE heatmap presented in Figure 3 suggests that a woman aged 50 to 60 years old with BMI 30, SBP 160 mm Hg, and HbA_1c_ 10% can expect to live an additional 3.0 years by reducing her SBP to 120 mm Hg, and can gain 1.2 years through reducing BMI to 25. In addition, findings from economic evaluations^35^ showed SBP control is cost-saving from a public health perspective. The relatively lower cost of antihypertensive medications and the established strong causal relationship between SBP and macrovascular complications^36^ make SBP control high clinical and economic value.

The benefit associated with treatment goal achievement declined sharply as the patient aged. For example, for a male patient aged 50 to 60 years with BMI 35, SBP 160 mm Hg, HbA_1c_ 8%, and LDL-C 130 mg/dL, reducing BMI from 35 to 30 was associated with an additional 1.4 years of LE. However, for a male patient aged 70 to 80 with the same levels of biomarkers, reducing BMI to 30 kg/m^2^ was only associated with an additional 0.6 years of LE. This finding emphasizes the importance of biomarker control at an earlier age. It also highlights the potential need for a trade-off between life quality and treatment for elderly patients when the benefit of biomarker control is limited. In addition, our estimation was based on the assumption that goal achievement was maintained for a lifetime. Individuals who met their goal at first but failed to maintain the level of biomarkers had lower benefits from our estimation.

Leal and colleagues^3^ used data from the UKPDS, with mortality rated between 1977 and 1997, to evaluate the LE gains associated with modifiable biomarker control in the UK population.^12^ Our study, on the other hand, used the most updated data (NHANES 2010-2016) to calibrate the BRAVO model to the modern mortality rates. The present estimates are higher than the estimations in the study by Leal et al^3^ for each of the 4 biomarkers. For a person with T2D and standard biomarker levels, the estimated LE was 75 to 80 years based on the study by Leal et al,^3^ and 80 to 85 years based on the BRAVO simulation. Advancements in medical technology and improvement in public health and health care systems in the last 3 decades could explain the LE gain.

The LE heatmap (Figure 3) was designed as a reference tool to support shared decision-making between clinicians and patients. Clinicians can easily locate the cell and the associated estimated life-years corresponding to a patient’s age, sex, and current biomarker values at the point of care. The clinician can then assess the potential gains in LE over a set of treatment goal options, and then show the patient how many additional life-years the patient could achieve by following the treatment plan on the heatmap. This intuitive method provides a tangible platform for the patient to visualize the benefit of the treatment, and thus can enhance the shared decision-making process and potentially improve patients’ motivation for treatment compliance. We plan to develop a smartphone application to allow more flexibility in specifying biomarkers and more patient characteristics, using the same approach used in this study.

### Limitations

Our study has several limitations. First, SGLT2 inhibitors were not included in the ACCORD trial. The benefits of HbA_1c_ reduction through SGLT2 inhibitors may be larger than we estimated, as evidence shows that SGLT2 inhibitors may provide additional cardiological protection in addition to HbA_1c_ control.^37^ However, most of this evidence is from people with established cardiovascular disease, which is not the target population of this study. We excluded patients with a history of cardiovascular disease, because for secondary prevention in patients with established cardiovascular disease, detailed drug-specific recommendations (eg, SGLT2 inhibitors for congestive heart failure prevention in people with T2D) are often recommended rather than simple biomarker control.^38^ Second, our estimations are limited by the projection accuracy of the BRAVO simulation model. In this study, the BRAVO model had an RB as low as 2% when projecting 30-year mortality against a nationally representative sample in the US even before the calibration process. Such high accuracy is due to the previous 2 rounds of extensive model validation and calibration, using data from 18 international clinical trials.^14,15^ We therefore believe that the estimations generated using the BRAVO simulation model have good scientific validity. Third, we were unable to distinguish type 1 diabetes from T2D because self-reported diabetes status in NHANES does not differentiate diabetes type. However, as more than 90% of US adults with diabetes have T2D,^39^ this bias will have limited consequences for our estimations. Fourth, the LE gain associated with a single biomarker reduction in clinical practice is likely to be even larger than our estimation because reductions in single biomarkers often lead to reductions in other factors simultaneously. A more advanced model that captures the associated spillover effect on other factors associated with risk when controlling a single biomarker would improve the accuracy of the estimation. Fifth, end-stage kidney disease was not selected as a variable in the BRAVO model. This could potentially lead to an over estimation of LE. However, considering the incidence of projected end-stage kidney disease is low, this overestimation issue would be minor. In addition, many other factors, such as triglyceride levels, may also play important roles in determining patients’ LE but are not included in the study. Further research is warranted to expand this analysis to a broader range of risk factors.

## Conclusions

We estimated the potential gains in LE associated with improvement in biomarkers, finding that improving each of the 4 biomarkers toward the recommended levels was associated with gains in LE, although the pattern and magnitude differed between them and according to patients’ characteristics (eg, age). Our findings can be used by clinicians and patients in selecting optimal treatment goals, to motivate patients in achieving them, and to measure potential health benefits for interventions and programs to improve diabetes care in the US.

## Notes

Supplement. **eAppendix.** Model Calibration **eTable 1.** Demographic Table for NHANES (2009-2010) **eTable 2.** Results Table for Model Performance **eTable 3.** Demographic Table for NHANES (2015-2016) **eFigure 1.** Additional Life-Years Gained From Quitting Smoking **eFigure 2.** The BRAVO Model’s Simulation Flow Chart **eFigure 3.** Life-Expectancies Associated With Different Levels of BMI, HbA_1c_, SBP, and LDL **eTable 4.** Risk Reductions in Diabetes Complications Associated With Goal Achievement in a 40-Year Window
eReferences
