# Supplementary material for: Potential Gains in Life Expectancy Associated With Achieving Treatment Goals in US Adults With Type 2 Diabetes
Source: JAMA Netw Open. 2022 Apr 18;5(4):e227705. doi: 10.1001/jamanetworkopen.2022.7705 (PMC10292109; doi:10.1001/jamanetworkopen.2022.7705)
Supplement: Supplement. — eAppendix. Model Calibration eTable 1. Demographic Table for NHANES (2009-2010) eTable 2. Results Table for Model Performance eTable 3. Demographic Table for NHANES (2015-2016) eFigure 1. Additional Life-Years Gained From Quitting Smoking eFigure 2. The BRAVO Model’s Simulation Flow Chart eFigure 3. Life-Expectancies Associated With Different Levels of BMI, HbA1c, SBP, and LDL eTable 4. Risk Reductions in Diabetes Complications Associated With Goal Achievement in a 40-Year Window eReferences [file jamanetwopen-e227705-s001.pdf]

Supplemental Online Content

Kianmehr H, Zhang P, Luo J, et al. Potential gains in life expectancy associated with achieving treatment goals in US adults with type 2 diabetes. *JAMA Netw Open*. 2022;5(4):e227705. doi:10.1001/jamanetworkopen.2022.7705

eAppendix. Model Calibration

eTable 1. Demographic Table for NHANES (2009-2010)

eTable 2. Results Table for Model Performance

eTable 3. Demographic Table for NHANES (2015-2016)

eFigure 1. Additional Life-Years Gained From Quitting Smoking

eFigure 2. The BRAVO Model's Simulation Flow Chart

eFigure 3. Life-Expectancies Associated With Different Levels of BMI, HbA1c, SBP, and LDL

eTable 4. Risk Reductions in Diabetes Complications Associated With Goal Achievement in a 40-Year Window

eReferences

This supplemental material has been provided by the authors to give readers additional information about their work.

© 2022 Kianmehr H et al. *JAMA Network Open.*

eAppendix 1 Model Calibration

The model calibration was conducted through the following steps: 1) a nationally representative simulation sample for T2D was generated using the National Health and Nutrition Examination Survey (NHANES, 2009-2010) data. We extracted patients' risk profiles, including demographic characteristics (age, race-ethnicity, duration of diagnosed diabetes, sex, education attainment, and smoking status), clinical complications (chronic kidney disease and blindness), and biomarkers (A1c, SBP, LDL, and BMI), from the individuals with self-reported T2D diagnoses by a health care provider. We excluded those with a history of cardiovascular disease (i.e., myocardial infarction (MI), congestive heart failure (CHF), stroke, and angina) from our sample. We further categorized the simulation sample into six age groups: 51-55, 56-60, 61-65, 66-70, 71-75, 76-80. 2) we used the BRAVO simulation model to predict 5-year mortality rates for each of the six age groups based on individuals' risk profiles; 3) we linked NHANES data with the data from the National Death Index (NDI) to estimate the true 5-year mortality rates for the six age groups; 4) We compared observed and predicted 5-year mortality rates across the six age groups. The NDI data only tracked the mortality of NHANES participants till 2015. In order to have 5-year mortality, the latest data we can use are from NHANES 2009-2010, which is why this wave of NAHENS was selected for model calibration.

We also plotted the simulated cumulative mortality rates versus Kaplan–Meier curve for observed mortality rates from age 50 to 80 (i.e., 30 years), assuming constant annual mortality rates within each age group. A calibrator was estimated for the BRAVO model to minimize the average relative bias (RB) in the six age groups, defined as the ratio of the difference between predicted and observed mortality rates to the observed mortality rate. We first estimated the average RB (ARB) across six subgroups and then estimated the calibrator using the following formula: 1/(1+RB). NHANES sampling and design weights were applied throughout the analysis. The calibrated mortality risk was a product of the original estimated mortality risk and the value of the calibrator.

A total of 519 individuals were identified from NHANES (2009-2010) for the model calibration. Demographic characteristics of the calibration sample are provided in eTable 1. Detailed model performance measurements are provided in eTable 2. Before calibration, the RB for 5-year mortality was estimated to be -22.4%, 63.3%, 9.8%, 11.5%, 25.5%, and -7.9% for age group 51-55, 56-60, 61-65, 66-70, 71-75, and 76-80, respectively. The RB for the 30-year mortality was 2.0%. After calibration, the RB for 5-year mortality was estimated to be -29.2%, 45.1%, -2.3%, 5.7%, 14.4%, and -16.6% for these age groups. The RB for the 30-year mortality was -4.5%. The average RB for 5-year mortality of the overall simulation samples was 13.3% before calibration and reduced to 2.9% afterward.

© 2022 Kianmehr H et al. *JAMA Network Open.*

Calibration results indicated that the BRAVO diabetes model produces more accurate estimations for older age groups than younger groups. When aiming at a longer period, such as 30-years, the BRAVO diabetes model is capable of generating estimations with RB as low as 2.8%. These results highlighted the subpopulation and time windows for which the model is most appropriate. Since the focus of this study was to generate LE predictions, the simulation was conducted over a lifetime window for all individuals in the simulation population. For individuals from the younger age group, the simulation window was mostly over 20 to 30 years, under which the BRAVO diabetes model can predict accurately. For the older population, the BRAVO diabetes model can predict the mortality rates well regardless of the time-window. Thus, we believe the simulation results we produced in this study are scientifically valid.

© 2022 Kianmehr H et al. *JAMA Network Open.*

eTable 1. Demographic Table for NHANES (2009-2010)

	NHANES 2009-2010											
	Age=51-55		Age=56-60		Age=61-65		Age=66-70		Age=71-75		Age=76-80	
Characteristics	Proportion (%) or mean	SE	Proportion (%) or mean	SE	Proportion (%) or mean	SE	Proportion (%) or mean	SE	Proportion (%) or mean	SE	Proportion (%) or mean	SE
**Age (years)**	53.4	0.3	58.0	0.3	63.2	0.2	68.0	0.2	72.7	0.2	79.1	0.1
**Gender**												
Female	47.2	8.5	53.7	9.1	44.9	6.5	50.3	7.2	57.8	6.0	53.6	5.3
Male	52.8	8.5	46.3	9.1	55.1	6.5	49.7	7.2	42.2	6.0	46.4	5.3
**Race**												
White	43.7	8.8	52.8	8.8	71.7	4.9	58.8	6.7	66.3	5.3	73.3	4.1
African American	25.8	6.0	21.0	5.6	9.0	2.3	14.3	3.4	13.8	3.3	14.4	3.0
Hispanic	19.0	4.4	17.7	4.3	12.0	2.2	12.1	2.6	11.9	2.8	7.2	2.0
Others	11.5	6.3	8.4	5.9	7.3	3.7	14.8	5.5	8.0	3.6	5.1	2.4
**Education**												
College graduates and above	60.0	7.7	39.0	9.1	51.5	6.5	50.3	7.2	39.7	6.1	36.5	5.2
Lower than college	40.0	7.7	61.0	9.1	48.5	6.5	49.7	7.2	60.3	6.1	63.5	5.2
**Current smoker**												
No	71.6	7.4	86.6	5.9	76.8	5.8	86.3	4.7	92.7	3.0	95.1	2.0
Yes	28.4	7.4	13.4	5.9	23.2	5.8	13.7	4.7	7.3	3.0	4.9	2.0
**Body mass index (BMI)**												
18–25 (normal weight)	19.8	4.5	5.6	2.6	6.2	3.1	11.2	4.5	12.9	4.1	19.8	4.5
25–30 (overweight)	33.7	5.1	18.9	8.0	24.9	5.4	18.2	5.1	29.0	5.5	33.7	5.1
>30 (obese)	44.5	5.5	75.5	8.2	68.9	5.9	70.5	6.3	58.1	6.1	44.5	5.5
Mean	34.4	1.0	34.4	1.0	33.4	0.8	34.3	1.0	32.6	0.9	29.5	0.6
**Time since diagnosis (years)**												
<5	23.0	5.0	36.3	9.3	37.3	6.5	39.2	7.1	19.5	4.8	23.0	5.0
5-10	33.0	5.4	16.9	4.9	17.9	5.1	24.6	6.1	20.9	5.0	33.0	5.4
10-15	10.5	3.4	28.2	9.5	20.0	5.4	17.1	5.8	24.2	5.3	10.5	3.4
>15	33.5	5.3	18.6	7.1	24.8	6.0	19.0	5.6	35.5	6.0	33.5	5.3
Mean	8.5	1.7	10.4	1.4	10.8	1.1	9.8	1.1	15.9	1.7	15.0	1.3
**HbA1c (%)**	7.2	0.3	7.2	0.2	7.3	0.2	6.9	0.1	7.0	0.1	6.8	0.1
**Systolic blood pressure (mmHg)**	129.3	2.8	127.8	2.3	126.9	2.3	130.3	2.6	137.1	2.5	135.9	2.2
**Low-density lipoprotein (mg/dl)**	102.8	6.0	106.5	8.2	101.9	7.2	94.2	6.7	102.9	6.3	87.2	4.0
**Cardiovascular history**												
Myocardial infarction	0.1	0.0	0.1	0.1	0.1	0.0	0.1	0.0	0.1	0.0	0.2	0.0
Angina	0.1	0.0	0.0	0.0	0.1	0.0	0.0	0.0	0.0	0.0	0.0	0.0
Congestive heart failure	0.0	0.0	0.1	0.0	0.1	0.0	0.1	0.0	0.1	0.0	0.2	0.0
Stroke	0.1	0.0	0.1	0.1	0.1	0.0	0.1	0.0	0.1	0.0	0.2	0.0
Revascularization surgery	0.0	0.0	0.1	0.1	0.0	0.0	0.1	0.0	0.1	0.0	0.1	0.0
**Mortality**	0.1	0.0	0.0	0.0	0.1	0.0	0.1	0.0	0.1	0.0	0.4	0.1

© 2022 Kianmehr H et al. *JAMA Network Open.*

eTable 2. Results Table for Model Performance

Age	Observed	Predicted		Calibrated	
	5-year Mortality*	5-year Mortality*	Relative Bias†	5-year Mortality*	Relative Bias†
51-55	5.1%	4.0%	-22.4%	3.6%	-29.2%
56-60	3.5%	5.7%	63.3%	5.1%	45.1%
61-65	8.9%	9.8%	9.8%	8.7%	-2.3%
66-70	11.8%	13.1%	11.5%	12.4%	5.7%
71-75	14.8%	18.6%	25.5%	16.9%	14.4%
76-80	39.0%	35.9%	-7.9%	32.5%	-16.6%
51-55	61.7%	62.9%	2.0%	59.0%	-4.5%
Average Relative Bias across six age groups			13.3%		2.9%

† Relative Bias was defined as the ratio of the difference between predicted and observed mortality rates to the observed mortality rate

* All numbers in these columns are 5-year mortality except the last row, which is the 30-year mortality for the age group 51-55.

© 2022 Kianmehr H et al. *JAMA Network Open.*

eTable 3. Demographic Table for NHANES (2015-2016)

	NHANES 2015-2016					
	Age=51-60		Age=61-70		Age=71-80	
Characteristics	Proportion (%) or mean	SE	Proportion (%) or mean	SE	Proportion (%) or mean	SE
**Age (years)**	55.7	0.3	65.4	0.2	76.4	0.3
**Gender**						
Female	50.4	4.4	45.9	3.8	42.6	4.5
Male	49.6	4.4	54.1	3.8	57.4	4.5
**Race**						
White	22.8	3.7	20.9	3.1	36.9	4.4
African American	24.4	3.8	25.6	3.3	13.9	3.1
Hispanic	40.9	4.4	41.3	3.8	31.1	4.2
Others	11.8	2.9	12.2	2.5	18.0	3.5
**Education**						
College graduates and above	45.7	4.4	43.6	3.8	41.8	4.5
Lower than college	54.3	4.4	56.4	3.8	58.2	4.5
**Current smoker**						
No	85.0	3.2	85.5	2.7	98.4	1.2
Yes	15.0	3.2	14.5	2.7	1.6	1.2
**Body mass index (BMI)**						
18–25 (normal weight)	11.8	2.9	12.4	2.5	17.2	3.5
25–30 (overweight)	29.1	4.0	32.5	3.6	39.7	4.5
>30 (obese)	59.1	4.4	54.4	3.8	43.1	4.6
Mean	33.7	0.8	31.6	0.5	29.8	0.5
**Time since diagnosis (years)**						
<5	43.2	4.6	23.6	3.3	15.7	3.4
5-10	22.9	3.9	24.2	3.3	25.2	4.1
10-15	15.3	3.3	19.4	3.1	22.6	3.9
>15	18.6	3.6	32.7	3.7	36.5	4.5
Mean	8.8	0.6	12.8	0.7	14.1	0.8
**HbA1c (%)**	8.0	0.2	7.5	0.1	7.2	0.1
**Systolic blood pressure (mmHg)**	131.2	1.7	134.6	1.3	139.0	1.9
**Low-density lipoprotein (mg/dl)**	115.5	5.8	94.6	3.4	88.3	3.6

© 2022 Kianmehr H et al. *JAMA Network Open.*

eFigure 1 Additional Life-Years Gained From Quitting Smoking

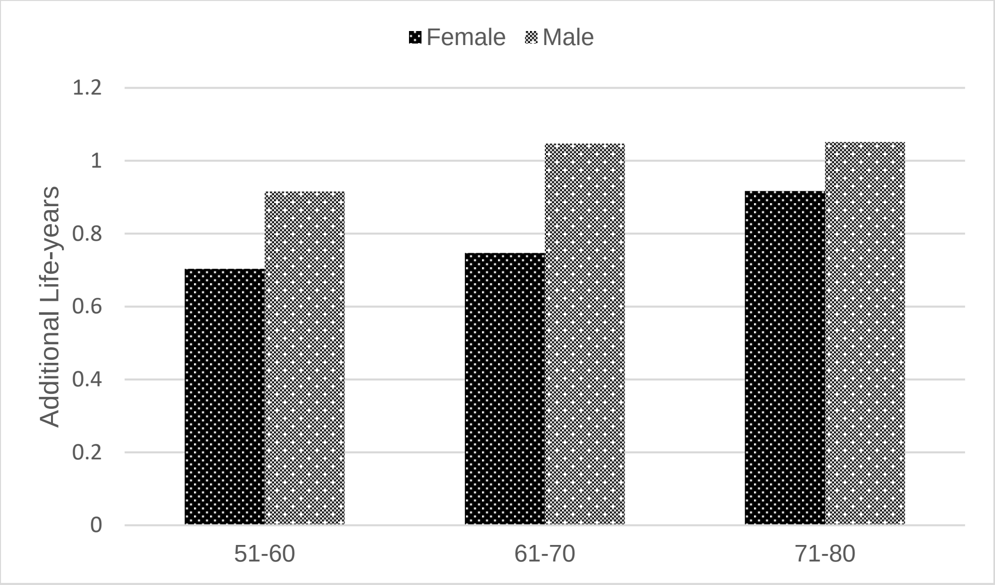

© 2022 Kianmehr H et al. *JAMA Network Open.*

eFigure 2. The BRAVO Model's Simulation Flow Chart

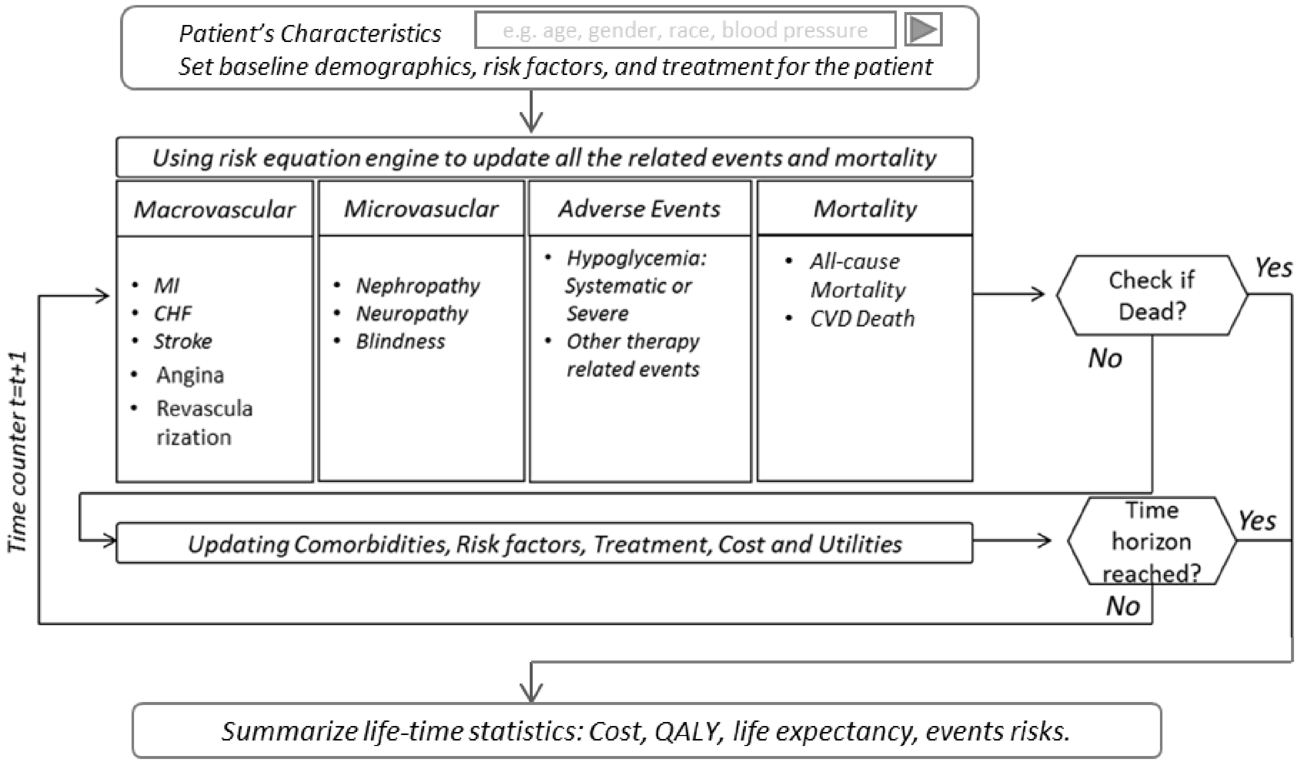

We've summarized the flow chart of the BRAVO model in eFigure 2. The simulation process was conducted at a personal level, instead of a traditional Markov-based cohort level. An annual cycle was adopted in the simulation, and each cycle, complications were examined at random order. To determine the occurrence of each complication, we estimated the probability of encountering each complication using the BRAVO risk equations.[1] This set of equations utilized the patient's characteristics and value of the biomarkers to estimate the likelihood of the complication. After that, the estimated probability was compared with a random number drawn from a uniform distribution (0-1). If the random number was lower than the estimated probability, we counted the person as encountering the corresponding complication. After all the complications were concluded, a check on death events was performed. If survived, the simulation process will be carried on to the next cycle, and this process kept going until a death event encountered, or the 5-year time horizon reached.

The BRAVO model contains a set of 17 intercorrelated risk equations for predicting the lifetime risk of macrovascular complications (MACE, myocardial infarction (MI), CHF, stroke, angina, revascularization), microvascular complications (chronic kidney disease (CKD), ESRD, blindness, neuropathy), hypoglycemia, mortality, and the progression of key biomarkers in diabetes (i.e., A1c,

© 2022 Kianmehr H et al. *JAMA Network Open.*

SBP, weight, low-density lipoprotein, and smoking). The model has been extensively validated and calibrated against 18 trials,[2] a nationally representative diabetes cohort,[3] and three SGLT2i trials: empagliflozin, dapagliflozin, and canagliflozin.[4] It is used by CDC[5] and the American Diabetes Association for policy/program evaluation[6], and by the Institute for Clinical and Economic Review (ICER) to evaluate the cost-effectiveness of GLDs.[7]

© 2022 Kianmehr H et al. *JAMA Network Open.*

eFigure 3 Life-Expectancies Associated With Different Levels of BMI, A1c, SBP, and LDL.

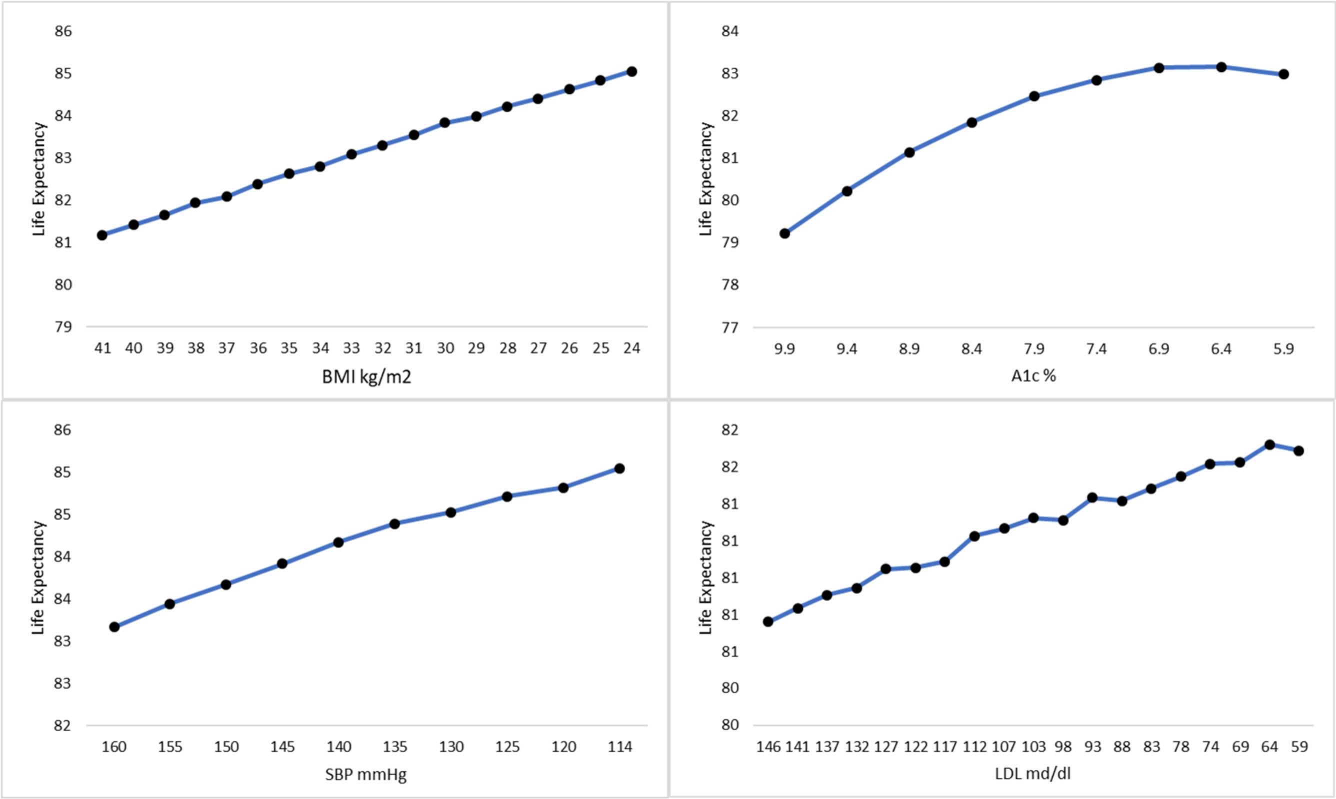

© 2022 Kianmehr H et al. *JAMA Network Open.*

eTable 4 Risk Reductions in Diabetes Complications Associated With Goal Achievement in a 40-Year Window

		Incidence Rates (# cases per 1000 person year)				Relative Risk		
	Event Types	Q4	Q3	Q2	Q1	Q3/Q4	Q2/Q4	Q1/Q4
HbA1c	Myocardial Infarction	373	334	291	247	0.90	0.78	0.66
	Stroke	479	291	218	157	0.61	0.46	0.33
	Hospitalization for CHF	343	369	335	293	1.07	0.98	0.85
	Hospitalization Angina	146	118	98	80	0.81	0.67	0.55
	End-stage Renal Disease	186	175	158	138	0.94	0.85	0.74
	Coronary revascularization procedure	886	938	892	818	1.06	1.01	0.92
	Blindness	725	649	572	489	0.90	0.79	0.67
	Nephropathy	1144	774	614	473	0.68	0.54	0.41
SBP	Myocardial Infarction	250	276	285	297	1.10	1.14	1.18
	Stroke	518	279	201	135	0.54	0.39	0.26
	Hospitalization for CHF	469	342	289	231	0.73	0.62	0.49
	Hospitalization Angina	81	85	84	89	1.05	1.04	1.09
	End-stage Renal Disease	194	144	125	103	0.74	0.64	0.53
	Coronary revascularization procedure	789	723	688	644	0.92	0.87	0.82
	Blindness	689	550	482	409	0.80	0.70	0.59
	Nephropathy	788	677	624	555	0.86	0.79	0.70
BMI	Myocardial Infarction	265	308	322	335	1.16	1.22	1.26
	Stroke	220	264	283	307	1.20	1.29	1.40
	Hospitalization for CHF	559	384	304	233	0.69	0.54	0.42
	Hospitalization Angina	136	120	108	95	0.88	0.79	0.70
	End-stage Renal Disease	158	179	188	202	1.14	1.19	1.28
	Coronary revascularization procedure	1035	997	847	650	0.96	0.82	0.63
	Blindness	521	603	645	683	1.16	1.24	1.31
	Nephropathy	707	815	866	929	1.15	1.22	1.31
LDL	Myocardial Infarction	477	372	318	268	0.78	0.67	0.56
	Stroke	593	395	310	239	0.67	0.52	0.40
	Hospitalization for CHF	415	427	424	428	1.03	1.02	1.03
	Hospitalization Angina	165	142	132	117	0.86	0.80	0.71
	End-stage Renal Disease	204	207	209	207	1.01	1.02	1.01
	Coronary revascularization procedure	1025	1050	1044	1051	1.02	1.02	1.03
	Blindness	802	715	659	607	0.89	0.82	0.76
	Nephropathy	1083	1024	990	952	0.95	0.91	0.88
Q: Quartile								

© 2022 Kianmehr H et al. *JAMA Network Open.*

eReferences

1. Shao H, Fonseca V, Stoecker C, Liu S, Shi L. Novel Risk Engine for Diabetes Progression and Mortality in USA: Building, Relating, Assessing, and Validating Outcomes (BRAVO). *PharmacoEconomics*. 2018;36(9):1125-1134. doi:10.1007/s40273-018-0662-1

2. Shao H, Yang S, Stoecker C, Fonseca V, Hong D, Shi L. Addressing Regional Differences in Diabetes Progression: Global Calibration for Diabetes Simulation Model. *Value in Health*. 2019;22(12):1402-1409. doi:10.1016/j.jval.2019.08.007

3. Kianmehr H, Zhang P, Pavkov ME, et al. 139-OR: ADA Presidents' Select Abstract: Potential Gains in Life Expectancy (LE) Associated with Achieving Treatment Goals in People with Type 2 Diabetes (T2D) in the U.S. *Diabetes*. 2021;70(Supplement 1). doi:10.2337/db21-139-OR

4. Shao H, Shi L, Fonseca VA. Using the BRAVO Risk Engine to Predict Cardiovascular Outcomes in Clinical Trials With Sodium–Glucose Transporter 2 Inhibitors. *Diabetes Care*. Published online April 17, 2020. doi:10.2337/dc20-0227

5. Shao H, Kianmehr H, Shi L, Fonseca V, Brown JD. 140-OR: Projected Impact of the Medicare Part D Senior Savings Model (SSM) on Diabetes-Related Health and Economic Outcomes among Insulin Users Covered by Medicare. *Diabetes*. 2021;70(Supplement 1). doi:10.2337/db21-140-OR

6. Shao H, Fonseca V, Furman R, Meneghini L, Shi L. Impact of Quality Improvement (QI) Program on 5-Year Risk of Diabetes-Related Complications: A Simulation Study. *Diabetes Care*. 2020;43(11):2847-2852. doi:10.2337/dc20-0465

7. Institute for Clinical and Economic Review. Tirzepatide for Type 2 Diabetes. Accessed September 11, 2021. https://icer.org/wp-content/uploads/2021/06/ICER_Type-2-Diabetes_Draft-Scope_063021.pdf

© 2022 Kianmehr H et al. *JAMA Network Open.*
